# Multiple Primary Malignancies in Head and Neck Cancer: A University Hospital Experience Over a Five-Year Period

**DOI:** 10.7759/cureus.17349

**Published:** 2021-08-21

**Authors:** Marta Vaz Batista, João Ulrich, Luís Costa, Leonor Abreu Ribeiro

**Affiliations:** 1 Medical Oncology, Hospital Prof. Doutor Fernando Fonseca, Lisboa, PRT; 2 Radiotherapy, Centro Hospitalar Universitário de Lisboa Norte, Lisboa, PRT; 3 Medical Oncology, Centro Hospitalar Universitário de Lisboa Norte, Lisboa, PRT

**Keywords:** field cancerization, head and neck cancer, multiple primary malignancies, second primary malignancy, head and neck neoplasms

## Abstract

Introduction

With an estimated incidence of 2%-4% per year, the development of a second primary malignancy (SPM) in patients with head and neck tumors (HNTs) is not a rare event. The present study aimed to (i) assess the frequency of SPMs in patients with HNTs treated in a university hospital over a five-year period and (ii) provide a demographic characterization of these patients.

Methods

Retrospective single-centre study of patients with more than one primary tumor (including at least one HNT) diagnosed between January 1, 2015, and December 31, 2019. Data were retrieved from patients’ clinical records and anonymized for analysis purposes.

Results

A total of 53 out of 824 (6.43%) patients with multiple primary malignancies were identified, 18 of which synchronous and 35 metachronous. The median follow-up was 25 months. Thirteen patients were diagnosed with more than one HNT. Forty patients were diagnosed with at least one HNT and one non-HNT. The most frequently diagnosed non-HNT SPMs were lung cancer (n=17) and esophageal cancer (n=8). The five-year survival rate was 53% for patients with multiple HNSCCs and 47% for patients with at least one non-HNT (log-rank p=0.729). The median overall survival was 14 months for synchronous and 58 months for metachronous SPMs (log-rank p=0.002).

Conclusion

Findings from this study highlight the importance of long-term follow-up of HNT patients for early detection of SPMs, increasing the chance of providing treatment with curative intent and improving patient outcomes and survival.

## Introduction

Head and neck tumors (HNTs) represent the seventh most common cancers worldwide, accounting for approximately 890.000 new cases and 450.000 deaths in 2018 [1]. HNTs comprehend a heterogeneity of malignancies arising in the head and neck anatomic region, including in the upper aerodigestive tract (oral cavity, nasal cavity, paranasal sinuses, pharynx, larynx, cervical esophagus), salivary glands, thyroid, associated lymph nodes, soft tissues, and bone [2]. The most common histological type is squamous cell carcinoma (SCC) and its variants [3].

The development of a second primary malignancy (SPM) in patients HNTs is not a rare event. Second tumors can be diagnosed either simultaneously/within six months of the index tumor − synchronous SPM − or more than six months after the index tumour − metachronous SPM. According to Warren and Gates criteria, diagnosis of an SPM requires (a) histologic confirmation of malignancy in both the index and second tumors; (b) that the two malignancies are anatomically separated by normal mucosa; and (c) exclusion of the possibility that the SPM is a metastasis from the index tumour [4]. 

The occurrence of SPMs has been explained by the concept of field cancerization, first described by Slaughter and colleagues in patients with oropharyngeal SCC [5]. This hypothesis considers that large areas of head and neck mucosa are affected by the same carcinogenic exposure, resulting in a wide field of premalignant disease with the same genetic alterations, which can give rise to multiple independent primary tumors [6]. 

Due to the clinical relevance of SPMs, follow-up of all patients with a primary HNT is recommended, not only to detect relapse and manage treatment-related toxicities but also for early detection of metachronous SPMs [7,8]. Indeed, the incidence of SPMs in patients with HNTs is estimated at 2%-4% per year, remaining relatively constant over time [9]. The present study aimed to (i) identify the frequency of SPMs in patients with HNTs treated in a university hospital over a five-year period and (ii) perform a demographic characterization of patients developing SPMs.

## Materials and methods

This was a single-centre retrospective study conducted in a university hospital in Lisbon. The study was approved by the local Ethics Committee. The clinical records of all patients included in the HNT multidisciplinary tumor board between January 1, 2015, and December 31, 2019, were reviewed.

Inclusion criteria were: (1) male or female patient ≥ 18 years old; (2) diagnosis of at least two malignant lesions confirmed by histopathological examination; (3) at least one of the malignant lesions must be an HNT; (4) patients cases presented in the HNT multidisciplinary tumor board between January 1, 2015, and December 31, 2019. 

Exclusion criteria were: (1) patients without histopathological confirmation malignancy; (2) second tumor malignancy suspected to be a metastasis; (3) no demographic and/or clinical information on patient´s electronic file. 

The following variables were collected from clinical records: year of birth; gender (male/female); smoking habits (active smoker - smokes every day and has smoked at least 100 cigarettes, former smoker - quit smoking at diagnosis but had smoked at least 100 cigarettes, or never smoker - smoked less than 100 cigarettes in the lifetime); alcohol consumption (moderate if <40 g/day, heavy if >40 g/day, or no alcohol consumption); the number of multiple primary tumors; SPM latency (synchronous/metachronous); therapeutic intervention for each tumor; treatment intent (curative/palliative); and date of death (if applicable). Data were analyzed using IBM SPSS Statistics 23®. Comparisons were made using chi-square and Fisher´s exact test. Kaplan-Meier curves were used to calculate survival.

## Results

A total of 824 patients were assessed in the hospital’s HNT multidisciplinary tumour board between January 1, 2015, and December 31, 2019. Of these, 53 patients (6.43%) had multiple primary malignancies, 18 of which were synchronous tumours and 35 metachronous tumours, and were included in the study. The median follow-up was 25 months. The demographic and clinical characteristics of the study population are shown in Table 1.

**Table 1:** Clinical characteristics of the study population. *All active smokers and former smokers with > 20 pack-year. **Light or moderate alcohol consumption < 40 g/day, heavy alcohol consumption >40 g/day. nr: number; CUP: cancer of unknown primary; HNT: head and neck tumor.

Clinical characteristics	nr.	%
Sex		
Male	50	94.3
Female	3	5.7
Smoking habits*		
Active smoker	20	37.7
Former smoker	19	35.8
Never smoker	1	1.9
Missing	13	24.5
Alcohol consumption**		
Moderate	8	15.1
Heavy	7	13.2
No alcohol consumption	2	3.8
Missing	36	67.9
Number of tumors/patient		
2	46	86.8
3	6	11.3
4	1	1.9
Latency		
Synchronous	18	34.0
Metachronous	35	66.0
Location of the primary malignancy		
Oral cavity	13	24.5
Pharynx	16	30.2
Larynx	20	37.7
Cervical cancer of unknown primary	2	3.8
Salivary gland	1	1.9
Thyroid	1	1.9
Location of the second primary malignancy − head and neck		
Oral cavity	5	9.4
Pharynx	2	3.8
Larynx	3	5.7
Cervical cancer of unknown primary	1	1.9
Thyroid	1	1.9
Location of the second primary malignancy − non-head and neck		
Lung	17	32.1
Esophagus	8	15.1
Colon	5	9.4
Hepatocellular carcinoma	3	5.7
Breast	2	3.8
Stomach	1	1.9
Anal	1	1.9
Skin	1	1.9
Central nervous system	1	1.9
Bladder	1	1.9
Sarcoma	1	1.9
Treatment intent (primary malignancy)		
Curative	46	86.8
Palliative	7	13.2
Treatment intent (second malignancy)		
Curative	37	69.8
Palliative	16	30.2
Group		
1 multiple HNTs	13	24.5
2 multiple tumors, at least one non-NHT	40	75.5

The median age at diagnosis of first malignancy was 61 (range 47−83) years. Most patients were male (n=50, 94.3%). Forty-six patients were diagnosed with two different malignancies, six with three, and one with four. For metachronous tumours, the median time for the development of SPMs was four years. Thirteen patients were diagnosed with more than one HNT. Forty patients were diagnosed with at least one HNT and one non-HNT. The most frequently diagnosed non-HNT SPMs were lung cancer (n=17) and esophageal cancer (n=8). Malignancies most frequently diagnosed in the same patient were larynx and lung cancer (n=8), oropharynx and esophagus cancer (n=4), and oral cavity and lung cancer (n=4). Table 2 summarizes the types of cancer found in the same patient. For 46 patients, the first diagnosed HNT was treated with curative intent. Seven patients were treated with palliative intent. SPMs were treated with curative intent in 37 patients.

**Table 2:** Types of cancer found within the same patient. nr.: number; CUP: cancer of unknown primary.

Tumor location 1	Tumor location 2	Tumor location 3	Tumor location 4	Nr.
Oral cavity	Oral cavity	Oral cavity		1
Oral cavity	Oral cavity			1
Oral cavity	Oral cavity			1
Oral cavity	Cervical CUP	Prostate		1
Oral cavity	Esophagus			1
Oral cavity	Larynx			2
Oral cavity	Breast			1
Oral cavity	Oropharynx			1
Oral cavity	Lung			4
Cervical CUP	Larynx			1
Cervical CUP	Lung			1
Salivary gland	Breast	Central nervous system		1
Hypopharynx	Hepatocellular carcinoma			1
Hypopharynx	Oesophagus			1
Hypopharynx	Lung			1
Hypopharynx	Bladder			1
Larynx	Anal			1
Larynx	Oral Cavity			2
Larynx	Colon			2
Larynx	Esophagus			2
Larynx	Skin			1
Larynx	Lung			7
Larynx	Lung	Oral cavity		1
Larynx	Central nervous system	Prostate	Renal	1
Larynx	Thyroid	Esophagus		1
Larynx	Oropharynx			1
Larynx	Sarcoma			1
Oropharynx	Oral cavity			1
Oropharynx	Hepatocellular carcinoma			2
Oropharynx	Colon			1
Oropharynx	Esophagus			4
Oropharynx	Stomach			1
Oropharynx	Lung			3
Thyroid	Colon			1

**Figure 1:**
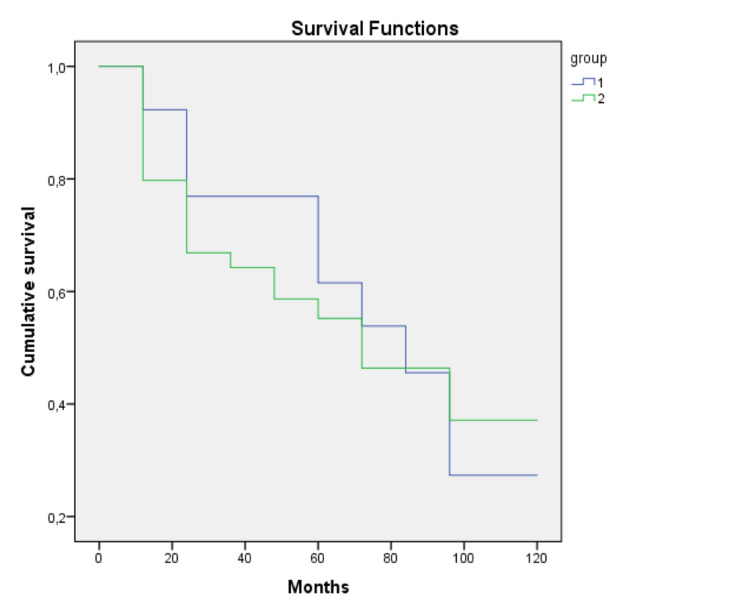
Overall survival in patients with only head and neck tumors and with at least one not head and neck tumor. Kaplan-Meier curves for overall survival for patients with second primary malignancies of the head and neck only (group 1) and with second primary malignancies in which at least one was not a head and neck tumor (group 2).

The overall survival (OS) of patients diagnosed with SPMs consisting only of HNTs (group 1) and patients diagnosed with SPMs in which at least one was not an HNT (group 2) was compared. Groups were comparable for age at first malignancy diagnosis and age at SPM diagnosis (p=0.470 and p=0.384, respectively). Figure 1 shows the Kaplan-Meier curves for OS in both groups. Five-year survival rate was 53% for patients with multiple HNTs and 47% for patients with at least one non-HNT (log-rank p=0.729). For most patients (21 out of 36), death was due to cancer progression. Five patients died of infectious causes (three with pneumonia and two with sepsis). For 10 patients, it was not possible to ascertain the cause of death.

**Figure 2:**
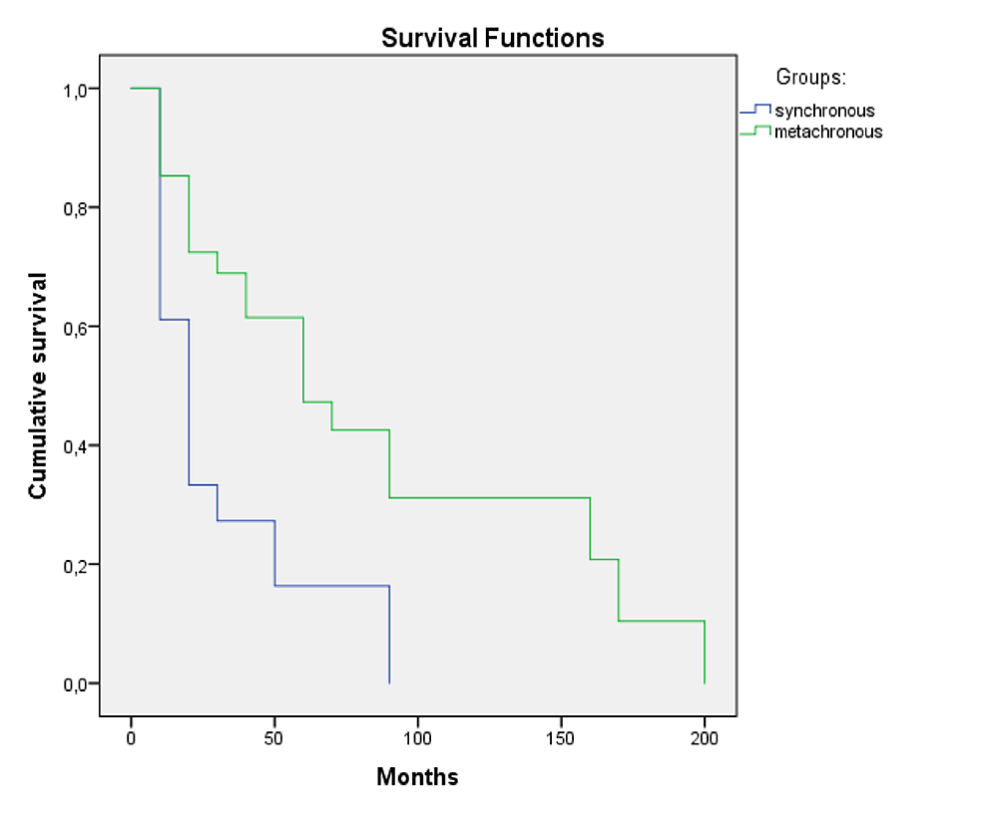
Overall survival for patients diagnosed with synchronous and metachronous tumors. Kaplan-Meier curves for overall survival for synchronous and metachronous tumors.

Median OS was 12.5 months for synchronous and 55 months for metachronous SPMs (log-rank p=0.033). Figure 2 depicts the Kaplan-Meier curves for OS in the two groups.

## Discussion

Within the five-year period in analysis, 6.43% of patients in a universe of 824 were identified with an SPM, which is consistent with previous reports [10,11]. From these patients, most (35 out of 53) were diagnosed more than six months after the primary malignancy, with a median time of SPM development of four years.

Lung cancer was the more frequent SPM, accounting for 32.1%, followed by head and neck (22.6%) and esophageal (15.1%) cancers. This data is consistent with previous reports, either from individual data [12] or pooled analyses [13]. Interestingly, the increased risk of lung cancer in patients with HNTs does not seem to be decreasing over the years, as opposed to head and neck and esophagus cancers [14].

As expected, most SPMs developed had acknowledged common risk factors with HNT [15]. Unfortunately, due to this study’s retrospective nature, relevant demographic data was missing, as alcohol consumption habits in most patients (67.9%) and smoking habits in 24.5%. Still, most patients for whom data was available had smoking and alcohol consumption habits.

Most malignancies were treated with curative intent (86.8% of first diagnosed cancers and 69.8% of SPMs), which is in line with previous reports [10]. Although not statistically significant, a trend was found towards inferior OS when SPMs were not HNTs, which was also reported by other authors [11]. Also consistently with previous studies, patients with metachronous SPMs had a better prognosis than those with synchronous SPMs [16]. Missing data and the small number of patients enrolled precluded the identification of a potential correlation between other clinical variables and increased risk of SPMs. 

Since this was a retrospective study, we were only able to analyse demographic data that were previous collected. Like previously said, smoking or drinking habits were missing in more than half of patients. We cannot also exclude a sample bias. As so, our results have to be seen as hypothesis generator.

Even so, our findings highlight the importance of long-term follow-up of these patients for early detection of SPMs, as recommended by European and American clinical practice guidelines [7,8].

## Conclusions

Follow-up visits should focus on early recognition of signs, symptoms, and/or imaging findings suggestive of SPMs. Early SPM detection increases the chance of providing treatment with curative intent and has the potential to improve patient outcomes. In our institution, most SPMs were treated with curative intent, and we believe that this might be explained by the regular follow-up politics adopted, that follows international guidelines. It is well acknowledged that preventive medicine has an important impact in the clinical outcomes of these patients, a fact that the study authors reinforce.

## References

[1] Bray F, Ferlay J, Soerjomataram I, Siegel RL, Torre LA, Jemal A (2020). Erratum: Global cancer statistics 2018: GLOBOCAN estimates of incidence and mortality worldwide for 36 cancers in 185 countries. CA Cancer J Clin.

[2] Chow LQ (2020). Head and neck cancer. N Engl J Med.

[3] Pathak J, Swain N, Patel S, Poonja L (2014). Histopathological variants of oral squamous cell carcinoma-institutional case reports. J Oral Maxillofac Pathol.

[4] Warren S, Gates O (1932). Multiple primary malignant tumors. A survey of the literature and a statistical study. Am J Cancer.

[5] Slaughter SP, Southwick HW, Smejkal W (1953). Field cancerization in oral stratified squamous epithelium; clinical implications of multicentric origin. Cancer.

[6] Tabor MP, Brakenhoff RH, Ruijter-Schippers HJ, Van Der Wal JE, Snow GB, Leemans CR, Braakhuis BJ (2002). Multiple head and neck tumors frequently originate from a single preneoplastic lesion. Am J Pathol.

[7] Machiels JP, René Leemans C, Golusinski W, Grau C, Licitra L, Gregoire V (2020). Squamous cell carcinoma of the oral cavity, larynx, oropharynx and hypopharynx: EHNS-ESMO-ESTRO Clinical Practice Guidelines for diagnosis, treatment and follow-up. Ann Oncol.

[8] (2021). National Comprehensive Cancer Network. Head and Neck Cancers (version 2.2021). https://www.nccn.org/login?ReturnURL=https://www.nccn.org/professionals/physician_gls/pdf/head-and-neck.pdf.

[9] León X, García J, López M, Rodrigues C, Gutierrez A, Quer M (2020). Risk of onset of second neoplasms and successive neoplasms in patients with a head and neck index tumour. Acta Otorrinolaringol Esp.

[10] Ng SP, Pollard C 3rd, Kamal M (2019). Risk of second primary malignancies in head and neck cancer patients treated with definitive radiotherapy. NPJ Precis Oncol.

[11] Kim SY, Roh JL, Yeo NK, Kim JS, Lee JH, Choi SH, Nam SY (2007). Combined 18F-fluorodeoxyglucose-positron emission tomography and computed tomography as a primary screening method for detecting second primary cancers and distant metastases in patients with head and neck cancer. Ann Oncol.

[12] León X, Del Prado Venegas M, Orús C, Kolañczak K, García J, Quer M (2005). Metachronous second primary tumours in the aerodigestive tract in patients with early stage head and neck squamous cell carcinomas. Eur Arch Otorhinolaryngol.

[13] Chuang SC, Scelo G, Tonita JM (2008). Risk of second primary cancer among patients with head and neck cancers: a pooled analysis of 13 cancer registries. Int J Cancer.

[14] Jégu J, Binder-Foucard F, Borel C, Velten M (2013). Trends over three decades of the risk of second primary cancer among patients with head and neck cancer. Oral Oncol.

[15] Rothman KJ (1978). Epidemiology of head and neck cancer. Laryngoscope.

[16] Priante AV, Castilho EC, Kowalski LP (2011). Second primary tumors in patients with head and neck cancer. Curr Oncol Rep.

